# Lent-On-Plus Lentiviral vectors for conditional expression in human stem cells

**DOI:** 10.1038/srep37289

**Published:** 2016-11-17

**Authors:** Karim Benabdellah, Pilar Muñoz, Marién Cobo, Alejandra Gutierrez-Guerrero, Sabina Sánchez-Hernández, Angélica Garcia-Perez, Per Anderson, Ana Belén Carrillo-Gálvez, Miguel G. Toscano, Francisco Martin

**Affiliations:** 1 Genomic Medicine Department. GENYO, Centre for Genomics and Oncological Research, Pfizer-University of Granada-Andalusian Regional Goverment, Parque Tecnológico Ciencias de la Salud, Av. de la Ilustración 114, 18016 Granada, Spain; 2 LentiStem Biotech. GENYO, Centre for Genomics and Oncological Research, Pfizer-University of Granada-Andalusian Regional Goverment, Parque Tecnológico Ciencias de la Salud, Av. de la Ilustración 114, 18016 Granada, Spain

## Abstract

Conditional transgene expression in human stem cells has been difficult to achieve due to the low efficiency of existing delivery methods, the strong silencing of the transgenes and the toxicity of the regulators. Most of the existing technologies are based on stem cells clones expressing appropriate levels of tTA or rtTA transactivators (based on the TetR-VP16 chimeras). In the present study, we aim the generation of Tet-On all-in-one lentiviral vectors (LVs) that tightly regulate transgene expression in human stem cells using the original TetR repressor. By using appropriate promoter combinations and shielding the LVs with the Is2 insulator, we have constructed the Lent-On-Plus Tet-On system that achieved efficient transgene regulation in human multipotent and pluripotent stem cells. The generation of inducible stem cell lines with the Lent-ON-Plus LVs did not require selection or cloning, and transgene regulation was maintained after long-term cultured and upon differentiation toward different lineages. To our knowledge, Lent-On-Plus is the first all-in-one vector system that tightly regulates transgene expression in bulk populations of human pluripotent stem cells and its progeny.

Technologies allowing conditional transgene expression in human stem cells are fundamental not only to study gene function^1^,^2^ but also as potential tools for gene therapy^3^. The ideal inducible system must achieve transgene regulation without affecting the normal physiology of the target cell. Tetracycline-regulated gene expression systems (Tet-On or Tet-Off) have been used successfully for conditional gene expression in most stem cells types including human embryonic stem cells (hESCs)^4^,^5^,^6^,^7^, induced pluripotent stem cells (iPSCs)^8^,^9^ and mesenchymal stromal cells (hMSCs)^10^,^11^,^12^. However, most tetracycline-regulated systems require a tetracycline-dependant-transactivator containing the activating domain of the herpes virus simplex viral protein 16 (*VP-16*) linked to the TetR repressor (tTA for Tet-Off or rtTA for Tet-On systems). The presence of this transactivating domain can make these proteins toxic due to the sequestration of transcription factors required for cell growth (squelching)^13^,^14^,^15^. In addition, binding of the chimeric TetR-VP16 protein to *pseudo-TetO* sites can trans-activate non-target cellular genes^16^,^17^ causing unpredicted side effects. Similar consequences have also been reported with other transcriptional activators such as the Cre-recombinase and its variant CreER^18^,^19^,^20^. Therefore, even though the tTA(rtTA)/tetO and Cre/*loxP* systems are useful tools for conditional transgene expression, they have the potential to influence cellular physiology.

Another major obstacle for the wider application of most conditional systems is the general requirement of drug selection to generate drug-responsive clones that can regulate transgene expression. The generation of regulatable stem cells clones is not always possible (i.e. hMSCs, HSCs) and, when possible, is time-consuming and labor-intensive. In this direction, efficient genetic manipulation of stem cells is a critical aspect to achieve direct transgene regulation. The gene delivery system must achieve stable expression of the regulator and long-term regulation of the transgene in target stem cells and in its progeny. The main hurdles to achieve this goal in stem cells are the low efficiency of gene delivery methods and the strong silencing of the transgenes^21^. In this direction, lentiviral vectors (LVs) represent an ideal tool because they integrate into the host genome, can accommodate multiple promoters and transgenes^22^,^23^ and are highly efficient at transducing stem cells including hematopoietic stem cells (HSCs)^24^, hMSCs^25^ and pluripotent stem cells (ESCs and iPSC)^26^,^27^.

However, although LVs are one of the most efficient systems to achieve stable transgene expression in stem cells, they are also prompt to transgene silencing^28^,^29^,^30^. Both the promoter expressing the regulator and the inducible promoter expressing the transgene can be silenced during stem cells expansion and/or differentiation^30^,^31^,^32^,^33^. Several approaches have been used to improve stability of LVs such as the use of human promoters^34^,^35^ or the incorporation of insulators^33^,^36^,^37^. The insulators are based on naturally occurring DNA elements that form functional boundaries between adjacent chromatin domains and play a role in shielding certain genes from other regulatory domains present on its proximity. In this direction, we recently developed a chimeric insulator (Is2) based on the chicken β-globin locus control region hypersensitive site 4 (HS4) and a synthetic scaffold/matrix attachment region (SAR2). The Is2 element was able to enhance expression and to avoid silencing of LVs in hESCs during expansion and upon differentiation toward the hematopoietic linage^33^.

Our group has previously described an all-in-one regulated lentiviral vector (CEST) based on the original TetR repressor, that allowed the generation of Dox-regulated cell lines, including primary human fibroblasts (HFF) and human MSCs (hMSCs) by repression of the strong CMV promoter. However the CEST LVs was unable to regulate transgene expression in pluripotent stem cells and required multiple integrations per cell in order to achieve regulation in 293 T and hMSCs^22^. In the present study, we have developed the all-in-one Lent-On-Plus LV systems able regulate transgene expression in pluripotent stem cells. This system is based on the original TetR repressor, only requires one copy vector per cell and the regulation is maintained over time in culture and upon differentiation.

## Results

### Development of an all-in-one LVs system with lower leaking and enhanced inducibility: The Lent-On-Plus system

**Figure 1:**
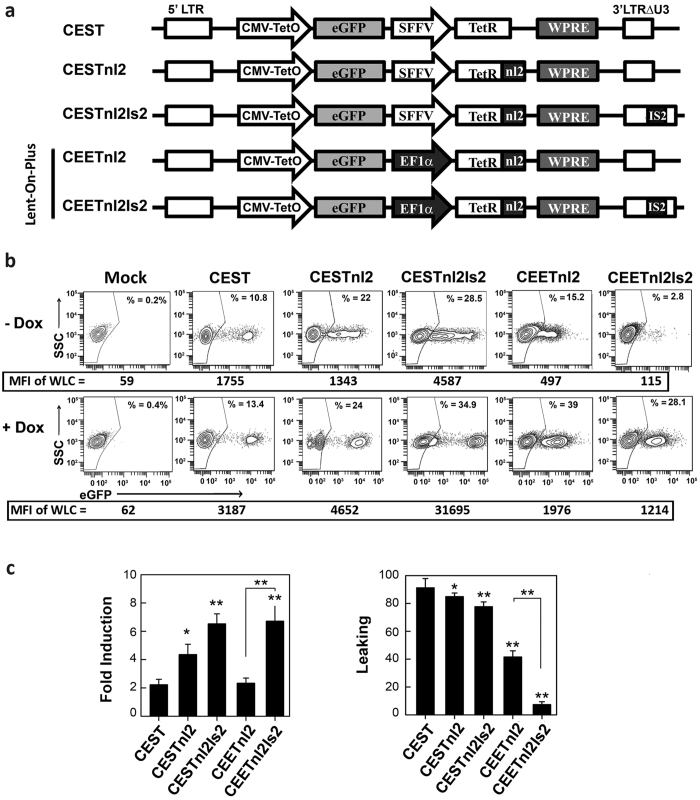
Second generation all-in-one LVs achieve tight transgene regulation in 293 T cells with just one copy integration/cell. (**a**). Schematic representation of the CEST LV^22^ (top) and the new all-in-one Tet-On LVs developed in this study (CESTnl2, CESTnl2Is2 and the Lent-On-Plus LVs: CEETnl2 and CEETnl2Is2). CMV (CMV-TetO); eGFP (enhanced green florescence protein); SFFV (spleen focus forming virus LTR promoter); hEF1α (human eukaryotic translocation elongation factor α1 promoter); TetRnl2 (TetR repressor incorporating the nuclear localization signal of the glucocorticoid receptor: see Fig. S1 for details). (**b**) Representative plots showing eGFP expression profiles of untransduced 293 T (Mock) and 293 T cells transduced with the different LVs (as indicated at the top of each plot) in the absence (top) or presence (bottom) of Dox (0.1 μg/ml). A MOI = 0.3 was used to keep the percentage of eGFP+ cells below 30% (in order to keep transduced cells with only one LV integration). The gates of the eGFP+ populations were set to 0.2–0.4% of eGFP+ cells in the untransduced population (Mock; left plots) and subtracted to the % obtained under the different vectors and conditions for the analysis. The percentage (%) of the eGFP+ population (used to measure leaking) are shown in each plot. The MFI of the whole living cells (WLC) (used to measure fold induction) are indicated at the bottom of each plot (**c**). Graphs showing fold induction (left) and leaking (right) of the different LVs in 293 T. Fold induction and leaking were calculated as indicated in M&M. To measure leaking, the background (% of eGFP+ of Mock cells) were subtracted to the % of the eGFP+ under the different conditions. Values represent mean +/− standard error of the mean of at least four separate experiments (*p < 0.05; **p < 0.01). Asterisks indicate significance related to CEST (on top of the bars) or significance between the CEETnl2 and CEETnl2Is2 (as indicated in the Figure)

In order to generate Tet-On all-in-one LVs that tightly regulate transgene expression in human stem cells we have constructed several LVs harboring different modifications (Fig. 1a). All the constructs express eGFP through the CMV-TetO promoter but have different backbones configurations to increase TetR levels in the nuclei, to avoid promoter silencing and to shield the LVs from enhancers present nearby the integration sites. The original TetR proteins translocate poorly to the nucleus^38^, a crucial step to achieve transcriptional repression. We initially tested two different nuclear localization signals and selected the TetRnl2 as the best repressor to be used in our LV system (Fig. S1).

Next, we constructed four all-in-one LVs harboring different promoters and insulator combinations (Fig. 1a); The CESTnl2 and CEETnl2 LVs express the TetRnl2 through the Spleen Focus Forming Virus (SFFV) and the human eukaryotic translocation elongation factor 1α (hEF1α) promoters respectively. The CESTnl2Is2 and CEETnl2Is2 LVs have the same backbone as the CESTnl2 and CEETnl2 respectively but including the insulator Is2^33^. The LVs expressing the TetR through the hEF1α promoter (CEETnl2 and CEETnl2Is2) were named Lent-On-Plus. The fold induction [MFI (+Dox)/MFI (−Dox)] and leaking ([%eGFP+(−Dox) *100/%eGFP+(+Dox)] of the different LVs were tested first in 293 T using a multiciplicity of infection (MOI) of 0.3. At this MOI most of the transduced cells harbored only one vector integrated in their genome, a circumstance where the original CEST had high leaking and poor fold induction(22 and Fig. 1b,c).

Expression of the TetRnl2 through the hEF1α promoter in 293 T cells decreased the leaking of the vectors in the absence of Dox (Compare CESTnl2 versus CEETnl2 in Fig. 1b, top plots and Fig. 1c, right graph) but also reduced the fold induction (Compare CESTnl2 versus CEETnl2 in Fig. 1c, left graph). It is worth mentioning that, In the presence of Dox, the CEETnl2 LV expressed lower eGFP levels compared to the CESTnl2 LV (Fig. 1b; MFI of the whole living cells (WLC) of CESTnl2 = 4652 versus CEETnl2 = 1976).

In spite of the improvements in leaking and fold induction of the CEETnl2 and CESTnl2 LVs, both vectors still presented high leaking and poor fold induction. Insulators, such as the chicken hypersensitive site 4 (cHS4)^39^ can shield integrative vectors from nearby regulatory domains favoring a more autonomous regulation and also act as barriers to avoid silencing^40^,^41^. SARs/MARs elements have also been incorporated into retroviral vectors improving their transgene expression and preventing promoter inactivation^42^,^43^,^44^. We have previously constructed the Is2 insulator (combining the HS4-650 and a synthetic SAR element) that was able to enhance expression and avoid silencing of LVs in hESCs^33^. We hypothesized that the inclusion of this element in the CESTnl2 and CEETnl2 LVs could improve their performance.

As expected, the CESTnl2Is2 LV showed improved expression levels compared to their Is2-negative counterpart (CESTnl2) in the presence of Dox (Fig. 1b; MFI of WLCs CESTnl2Is2 = 31695 versus 4652 in CESTnl2). The higher expression levels of the Is2-containing LVs rendered also a higher fold induction of these LVs (Fig. 1c, left graph). However, this enhancement of expression was not observed when the Is2 was incorporated in the CEETnl2 backbone (MFI = 1976 versus 1214 of CEETnl2 versus CEETnl2Is2 respectively). Yet, the fold induction of the CEETnl2Is2 LVs increased compared to the CEETnl2 LVs (Fig. 1c, left graph) due to the lower leaking in the absence of Dox (Fig. 1c, right graph). In terms of leaking, the effect of the Is2 element has the same tendency in both backbones but is significant only on the CEETnl2 LVs.

Leaking of a regulatable system can be analyzed in different ways. For simplicity, we have used the formula; leaking = [%eGFP+(−Dox) *100/%eGFP+(+Dox) that generate values from 0 (no leaking) to 100 (absence of regulation). However, this analysis does not discriminate between partially-responsive cells from those cells that do not respond to Dox. We have therefore reanalyzed the leaking using an alternative formula; leaking = [% eGFP^+^ (−Dox)/%eGFP^+^(+Dox)] × MFI eGFP^+^ cells (−Dox)] to take into account the expression levels of the transduced cells in the absence of Dox (Fig. S2). This analysis confirmed that the nl2 signal reduced the leaking of the CEST LVs and that the Is2 element reduced leaking only in the CEET LV backbone.

All together these data indicate that only the CEETnl2Is2 LV was able to regulate transgene expression in 293 T harboring one vector copy number per cell (vcn/c). As a down side, we detected a reduction on the titer (2–3 fold) of the Is2-LVs compared to their Is2-negative counterparts (data not shown).

The fold induction parameter indicates the increment of eGFP expression of the total population upon the addition of Dox (see M&M for details). Since we kept transduction below 40% (to obtain cell populations harboring only one vector copy number per cell (vcn/c)) fold induction are underestimated. To study the potency of the system we further analyzed the inducibility of the Lent-On-Plus LVs (CEETnl2 and CEETnl2Is2) on cell populations that are 100% responsive to Dox (Fig. S3). These data confirmed the positive effect of the Is2 insulator on leaking (Fig. S3 CEETnl2 = 66.2 versus CEETnl2Is2 = 7.4) and fold induction (CEETnl2 = 5.7 versus CEETnl2Is2 = 38.8) in the Lent-On-Plus LVs (CEET backbone).

### The Is2+ Lent-On-Plus LVs efficiently regulate transgene expression in human mesenchymal stromal cells

**Figure 2:**
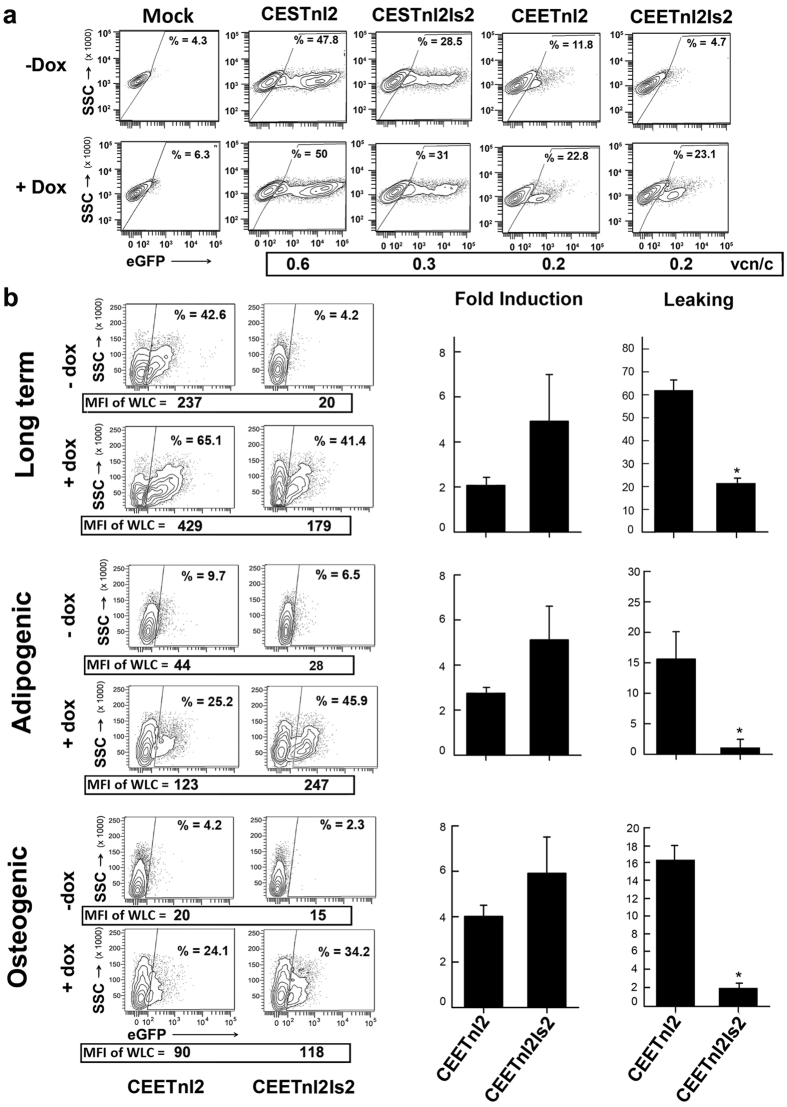
Performance of the different Tet-On LVs on hMSCs. (**a**) Representative plots showing eGFP expression profiles of untransduced hMSCs (Mock) and hMSCs transduced with CESTnl2, CESTnl2Is2, CEETnl2 and CEETnl2Is2 LVs in the absence (top) or presence (bottom) of Dox (0.1 μg/ml). Since hMSCs are more resistant to LVs transduction than 293 T cells, a MOI = 1 was used to keep the percentage of transduced hMSCs below 30%. Vector copy number per cell (vcn/c) is indicated at the bottom of each plot. The gates of the eGFP+ populations were set to have 4–6% of eGFP+ cells in the untransduced population. The percentage (%) of the eGFP+ population are shown in each plot (**b**) hMSCs transduced with the Lent-On-Plus (CEETnl2 and CEETnl2Is2) LVs where kept in culture for up to 40 days (top plots and graphs) or differentiated toward the adipogenic (middle plots and graphs) and osteogenic (bottom plots and graphs) lineages. Representative plots showing eGFP expression profiles in the absence or presence of Dox are shown at the left and graphs showing fold induction and leaking are shown at the right. The MFI of the whole living cells (WLC) (used to measure fold induction) are indicated at the bottom of each plot. The gates of the eGFP+ were set to have 0.6%, 5,9% and 1.7% of eGFP+ cells in the untransduced population of the long term, adipogenic and osteogenic cells respectively (Fig. S5). Values represent mean +/− standard error of the mean of at least three separate experiments (*p < 0.05)

We next tested the new inducible systems in hMSCs. We transduced hMSCs with the CESTnl2, CESTnl2Is2, CEETnl2 and CEETnl2Is2 LVs at a MOI = 1 and 10 days later analyzed for GFP expression in the presence or absence of Dox (Fig. 2a). As observed in 293 T cells, the expression of the TetRnl2 through the hEF1α promoter (Lent-On-Plus LVs) reduced the leaking of the LVs (Fig. 2a right top plots) even at low vcn/c. Importantly, the combination of the hEF1α promoter and the Is2 insulator blocked the expression of eGFP almost completely in the absence of Dox (Fig. 2a, compare untransduced (top-left plot) with CEETnl2Is2-transduced (top-right plot) hMSCs). On the contrary, the vectors expressing TetRnl2 through the SFFV promoter (CESTnl2 and CESTnl2Is2) had high leaking at low vcn/c (Fig. 2a, top-left plots). Interestingly, the CEETnl2 LVs express even lower TetR levels than CESTnl2 LVs in MSCs (Fig. S4) which indicates that the lower leaking of the CEETnl2 is not due to higher expression levels of the repressor but to the Lent-On-Plus backbone configuration. Based on these results we focused on the CEETnl2 and CEETnl2Is2 (Lent-On-Plus systems) for later studies.

Inducible systems should be able to maintain transgene regulation upon cellular expansion and/or differentiation. We therefore transduced hMSCs with the CEETnl2 and CEETnl2Is2 LVs and studied transgene regulation after expansion (Fig. 2b, Top panels) and differentiation toward the adipogenic (Fig. 2b, middle panels) and osteogenic (Fig. 2b, bottom panels) lineages (Fig. S5 shows the characterization of differentiated cells). In all cases, the CEETnl2Is2 had the lowest leaking and higher fold induction as can be observed in the representative plots (Fig. 2b, left) and in the graphs (Fig. 2b, right). It is remarkable that neither the long-term culture nor the differentiation of the hMSCs toward the adipogenic or osteogenic lineages influenced the low leaking and inducibility of the CEETnl2Is2 LVs in this cell type. As in 293 T cells, leaking of the different LVs were also re-analyzed to take into account the MFI. The new analysis showed similar conclusions but improved significance of some results (Fig. S6).

**Figure 3:**
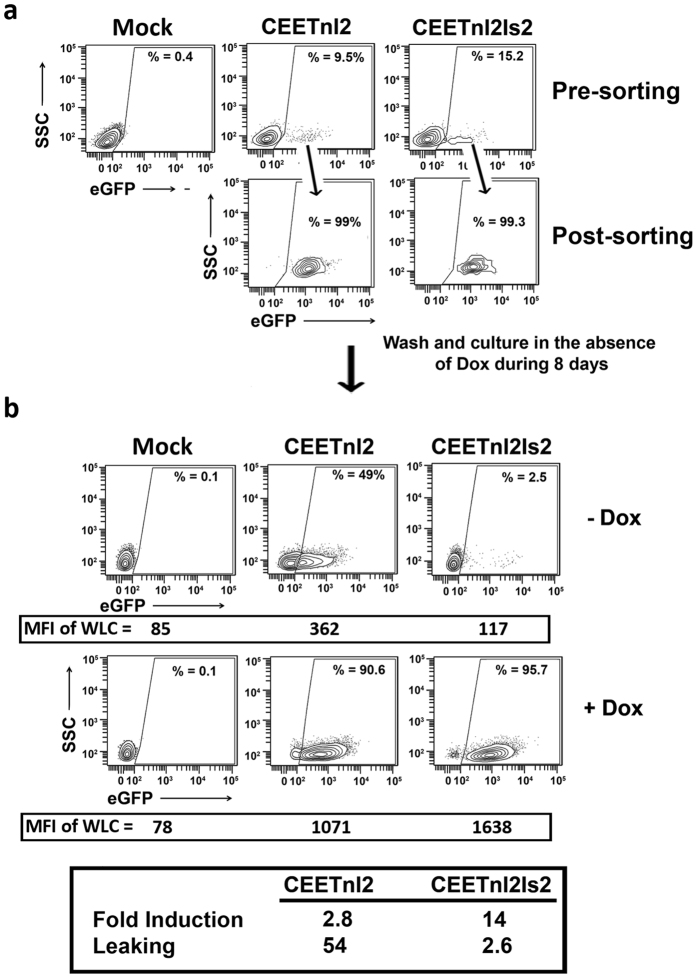
Performance of the Lent-On-Plus LVs on sorted hMSCs. (**a**) Plots showing eGFP expression before (top plots) and after (bottom plots) purification of the eGFP+ populations of CEETnl2 and CEETnl2Is2-transduced hMSCs using fluorescence-activated cell sorting (FACS) Aria II Flow Cytometer. A MOI = 0.4 was used to keep the percentage of transduced hMSCs below 15%. (**b**) After sorting, the hMSCs were kept in the absence of Dox for 8 days and then induced (+Dox) or not (−Dox) for additional 5 days and analyzed for eGFP expression. The Percentages (%) of eGFP+ cells in the different population are shown inside each plot. The MFI of the whole living cells (WLC) (used to measure fold induction) are indicated at the bottom of each plot. Fold induction and leaking of the CEETnl2- and CEETnl2Is2-transduced sorted populations are indicated in the table (bottom).

Finally we aimed to generate pure populations of CEETnl2 and CEETnl2Is2- cells harboring 1 vcn/c. hMSCs were transduced at low MOI to achieve 9–15% eGFP+ cells upon induction with Dox. Two weeks later eGFP+ hMSCs were sorted to a purity of 99% (Fig. 3a), cultured for 8 days in the absence of Dox and then analyzed for responsiveness to Dox (Fig. 3b). The results confirmed the positive effect of the Is2 insulator on leaking (CEETnl2 = 54 versus CEETnl2Is2 = 2.6) and fold induction (CEETnl2 = 2.8 versus CEETnl2Is2 = 14) in hMSCs. Since in these populations all cells contain only one vector integrated in their genome, the good fold induction obtained with the CEETnl2Is2 (14) represent the average dox response of one Lent-On-Plus LV integrated in hMSCs. Importantly, the leaking of the CEETnl2Is2 LVs remains very low (2.8) with only a minor increment in MFI of the WLC compared to the untransduced population (Fig. 3b: MFI of WLC Mock = 85 versus MFI of WLCs CEETnl2Is2 –Dox = 117)

### Generation of doxycycline-responsive pluripotent stem cells by the CEETnl2 and the CEETnl2Is2

**Figure 4:**
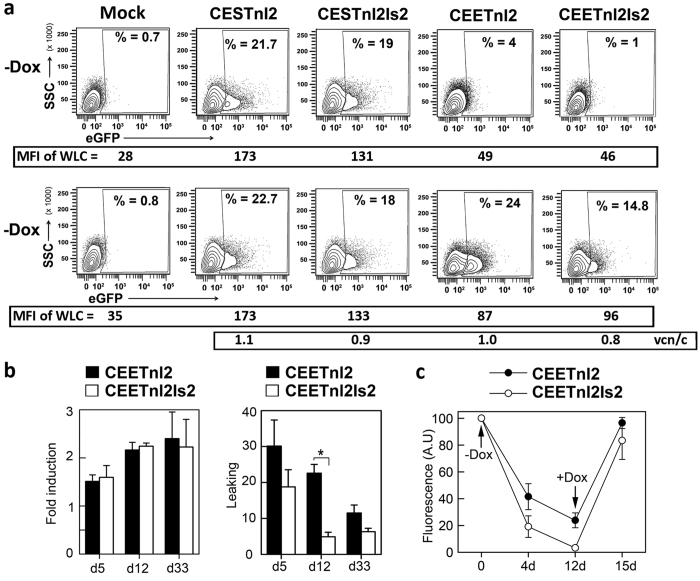
Lent-On-Plus (CEETln2 and CEETln2Is2) LVs efficiently generates Doxycycline-responsive hESCs without selection or cloning. (**a**) Representative plots showing eGFP expression profiles of hESCs (AND-1) control (Mock) and hESCs transduced with CESTnl2, CESTnl2Is2, CEETnl2 and CEETnl2Is2 LVs in the absence (top) or presence (bottom) of Dox (0.1 μg/ml). hESCs were transduced at MOI = 5 with the different LVs and analyzed 10 days later. The percentages (%) of the eGFP+ populations are shown in each plot. The MFIs of the whole living cells (WLC) are indicated at the bottom of each plot. The vcn/c of transduced cells are shown at the bottom of the figure. (**b**) Graphs showing fold induction (left) and leaking (right) of the CEETnl2 and CEETnl2Is2 LVs in hESCs at different time points during expansion (day 5, day 12 and day 33 post-transduction). Values represent mean +/− standard error of the mean of at least three separate experiments using AND-1 and H9 hESCs transduced at MOI = 5 with the different LVs (*p < 0.05). (**c**) Graph showing relative eGFP expression levels of CEETnl2 and CEETnl2Is2-transduced hESCs (AND-1) upon withdrawal (day 0) and addition (Day 12) of Dox along time in culture. Data represent fluorescence intensity relative to Day 0. Values represent mean +/− standard error of the mean of at least three separate experiments.

The generation of pluripotent stem cells expressing the desired transgene under the tight control of an inducer has been a very difficult task to achieve due to the low efficiency of existing delivery methods, the strong silencing of the transgenes and the side effects of the transactivators. We hypothesized that the use of insulated LVs expressing the TetRnl2 through stable promoters could overcome previous existing limitations to generate Dox-inducible hESCs. To investigate this possibility, we first tested the Dox-responsiveness of two hESCs lines (AND-1 and H9) transduced with the CESTnl2, CESTnl2Is2, CEETnl2 and CEETnl2Is2 LVs at MOI = 5 (Fig. 4). Neither, the CESTnl2 nor CESTnl2Is2 LVs could regulate transgene expression in hESCs (Fig. 4a, left plots). On the contrary, both Lent-On-Plus LVs expressing TetRnl2 through the hEF1α promoter (CEETnl2 and CEETnl2Is2) achieved low leaking (Fig. 4a; compare Mock versus CEETnl2- and CEETnl2Is2- transduced hESCs) and good transgene expression in the presence of Dox at low vcn/c (Fig. 4a, right plots and Fig. S7). However, it is important to highlight that not all transduced-hESCs will express eGFP in the presence of Dox as can be observed in Fig. 4a. Indeed, only around 20% of the hESCs express eGFP in spite of having an average of 1 vcn/c. This observation has been described previously and is probably due to transcriptional variegation of the integrated LVs. We next studied whether CEETnl2- and CEETnl2Is2-transduced hESCs maintained transgene regulation upon cellular expansion. We therefore cultured the inducible hESCs cells in the presence or absence of Dox for 33 days and analyzed their leaking and fold induction at different time points (Fig. 4b). Both vectors kept their Dox-responsiveness (fold induction) during time in culture without significant changes (Fig. 4b, left graph) and, as observed in hMSCs, the presence of the Is2 insulator reduced the leaking of the CEET LVs (Fig. 4b, rRight graph). However, contrary to what was observed in MSCs, the CEETnl2Is2 LVs have similar fold induction compared to the CEETnl2 LVs (Fig. 4b, left graph). We additionally showed that we can turn off and turn on transgene expression in the CEETnl2- and CEETnl2Is2-transduced hESCs by withdrawal or addition of Dox (Fig. 4c).

**Figure 5:**
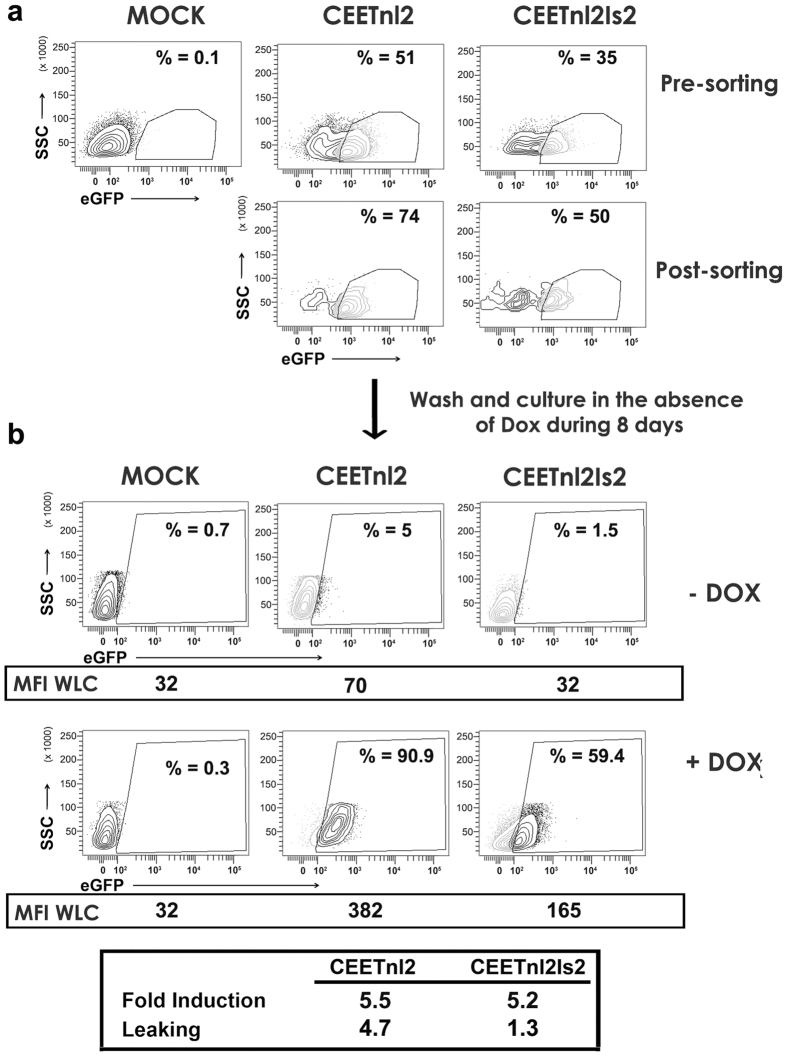
Performance of the Lent-On-Plus LVs on sorted hESCs. (**a**) Plots showing eGFP expression before (Pre-sorting) and after (Post-sorting) purification of the eGFP+ populations of CEETnl2 and CEETnl2Is2-transduced hESCs using fluorescence-activated cell sorting (FACS) Aria II Flow Cytometer. A MOI = 30 was used to achieve over 35% eGFP+ cells and good expression levels. (**b**) Sorted hESCs were kept in the absence of Dox for 8 days and then induced (+Dox) or not (−Dox) for additional 5 days and analyzed for eGFP expression. The Percentages (%) of eGFP+ cells are shown inside each plot. The MFI of the whole living cells (WLC) (used to measure fold induction) are indicated at the bottom of each plot. Fold induction and leaking of the CEETnl2- and CEETnl2Is2-transduced sorted populations are indicated in the table (bottom).

We next generated sorted CEETnl2- and CEETnl2Is2-transduced hESCs in order to study Dox responsiveness in a more pure population. hESCs were transduced at MOI = 30 and two weeks later, CEETnl2- and CEETnl2Is2-transduced hESCs were sorted to a purity of 74 and 50% respectively (Fig. 5a). Sorted cells were cultured for 8 days in the absence of Dox and Tra-1–81+/SSA3+ hESCs analyzed for responsiveness to Dox (Fig. 5b). The results corroborate the positive effect of the Is2 insulator on leaking (CEETnl2 = 4.7 versus CEETnl2Is2 = 1.3) but not in fold induction (CEETnl2 = 5.5 versus CEETnl2Is2 = 5.2).

### The hEF1α promoter is a key factor for regulation of the CEETnl2 and CEETnl2Is2 LVs

**Figure 6:**
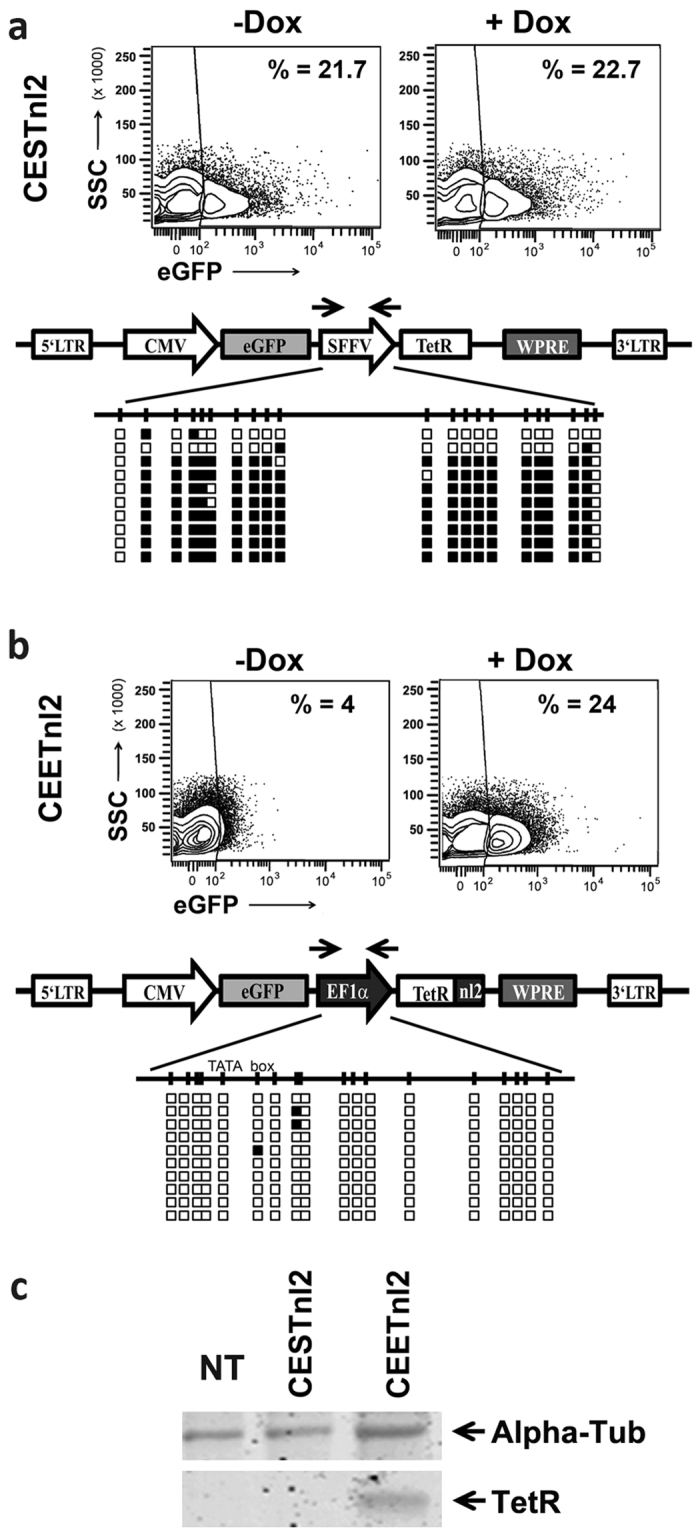
Methylation analysis of all-in-one Tet-ON LVs in hESCs. DNA from CESTnl2- (**a**) and CEETnl2- (**b**) transduced hESCs were extracted at day 15 post-transduction and converted with sodium bisulfate (see M&M for details). Plots showing Dox responsiveness of CESTnl2 and CEETnl2 at the time of lysis are shown at the top of (**a**,**b**) respectively. The converted DNA was subjected to PCR using the primers pair indicated in each figure and sequenced. The drawing below the LV schemes (bottom panels in (**a,b**)) represents the CpG islands contained in the promoter region that was analyzed. Black and white boxes represent methylated and unmethylated CpG respectively. It can be observed that SFFV is highly methylated in AND-1 (**a**) while hEF1α is highly resistant to methylation (**b**). (**c**) TetR expression is restricted to the CEET-transuced hESCs. hESCs (AND-1) transduced with the CESTnl2 and CEETnl2 were lysed and analyzed for TetR expression by Western Blot (see M&M for details).

Transgene silencing due to promoter methylation of integrative RVs has been a major problem for stable expression in hESCs. Several studies have shown that pluripotent cells can block expression of exogenous retroviral elements by complex defense mechanisms^45^. It has been previously described that the SFFV promoter is silenced through methylation in human pluripotent cells^29^ while the hEF1α promoter is highly resistant allowing stable transgene expression in these cell types^34^,^46^,^47^. We therefore analyzed the methylation profiles of the SFFV and hEF1α promoters in CESTnl2- and in CEETnl2-transduced hESCs (Fig. 6a,b respectively). As expected by the low leaking and good inducibility of the CEETnl2 (Fig. 6b, plots), the hEF1α promoter presented a very low degree of methylation (Fig. 6b, bottom panel). On the contrary, the SFFV promoter in the CESTnl2 LVs was highly methylated (>90%; Fig. 6a, bottom panel) which resulted in the lost of transgene regulation (Fig. 6a, top plots). Of note, the presence of Is2 insulator in the CESTnl2Is2 LVs was not able to avoid SFFV methylation (data not shown). We further corroborated the influence of promoter methylation on TetR expression levels by analyzing CESTnl2- and CEETnl2-transduced hESCs by western blot (Fig. 6c). Only the CEETnl2-transduced hESCs expressed detectable TetR protein as was expected by the absence of regulation in these cells and by the methylation of the SFFV promoter in the CESTnl2 LVs.

### Doxycycline responsiveness of the Lent-On-Plus LVs is maintained after differentiation of hESCs toward the mesenchymal and hematopoietic lineages

**Figure 7:**
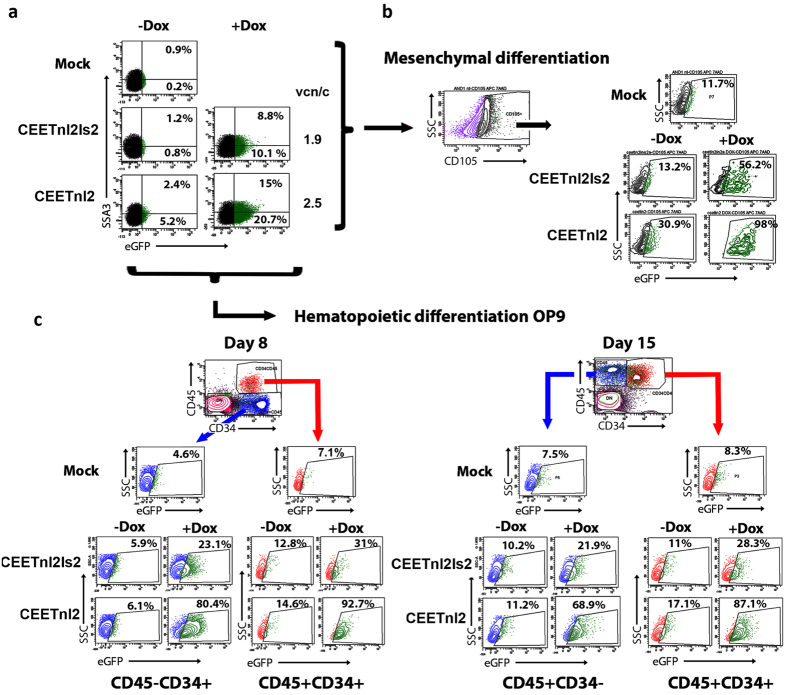
Transgene regulation of Lent-On-Plus (CEETnl2 and CEETnl2Is2) in mesenchymal and hematopoietic cells derived from hESCs. (**a**) AND-1 hESC were transduced with concentrated Lent-On-Plus (CEETnl2 and CEETnl2Is2) LVs. eGFP and SSA3 (a pluripotency marker) expressions were analyzed in the presence (left plots) or absence (right plots) of Dox (0.1 μg/ml). The gate of the eGFP+ populations were set to have 0.5–1-% of SSE3+eGFP+ cells in untransduced population (Mock; top plot). (**b**) Lent-On-Plus regulatable hESCs were differentiated to mesenchymal stromal cells (see M&M for details). At day 10 of differentiation Dox was added and eGFP expression levels analyzed 5 days later. The plots show eGFP expression levels in the CD105 population of untransduced (Mock; top plot), CEETnl2Is2-transduced (middle plots) and CEETnl2 (bottom plots) in the presence or absence of Dox as indicated at the top of the plots. (**c**) Lent-On-Plus regulatable hESCs were differentiated toward the hematopoietic lineage using the OP9 differentiation protocol (see M&M for details). The cells were maintained in the presence or absence of Dox from day 1 of differentiation and the eGFP expression analyzed in the different hematopoietic populations (CD45 − CD34+, CD45+CD34+ and CD45+CD34−) as indicated at the different days (Day 8; Top-Left plot and Day 15; Top-right plot). Background levels in untransduced hESCs were set between 4.6–8.3% for each population (arrows) at each day of differentiation (Mock, second top plots). The percentage of cells expressing eGFP in the presence or absence of Dox of the CEETnl2- and CEETnl2Is- transduced hESCs are shown inside each plot for each hematopoietic population.

Ideally, the inducible systems for pluripotent stem cells must be also able to maintain drug responses upon differentiation toward different cell types. We then analyzed whether the Lent-On-Plus systems (CEETnl2 and CEETnl2Is2) maintained the Dox responsiveness in mesenchymal and hematopoietic cells derived from transduced hESCs (Fig. 7).

hESCs transduced with the CEETnl2 and CEETnl2Is2 LVs at high MOI (vcn/c of 2.5 and 1.9 respectively) (Fig. 7a) were differentiated toward hMSCs-like cells as described previously^48^ (see M&M for details). At day 10 of differentiation, homogeneous fibroblast-like cells were obtained and at this point, Dox was added and eGFP expression levels analyzed in the CD105+ (a hMSCs marker) population (Fig. 7b). Our data showed that hMSCs-derived from CEETnl2-transduced hESCs had low leaking (compare Mock- with CEETnl2- and CEETnl2Is2-transduced cells in Fig. 7b) and a good expression levels after Dox addition (Fig. 7b, right plots). These results indicate that the expression of the TetRnl2 repressor through the hEF1α promoter was enough to achieve stable transgene regulation even after hESCs differentiation into hMSCs. The high titer of the CEETnl2 allowed high transduction efficiency of hESCs (Fig. 7a) achieving up to 98% eGFP+ cells upon hMSC differentiation (Fig. 7b). The CEETnl2Is2 LVs were less efficient (56%) due to the lower titer, but the CEETnl2Is2-hESCs derived hMSCs were more tightly regulated (lower leaking) than CEETnl2-hESCs derived hMSCs (Fig. 7b, upper versus lower plots; −Dox). Therefore, the presence of the Is2 insulator is required for a tight control of gene expression in hESCs-derived hMSCs which is in agreement to what was observed in hMSCs (Figs 2 and 3).

We finally differentiated CEETnl2- and CEETnl2Is2- transduced hESCs toward the hematopoietic lineage using the OP9 differentiation protocol as previously described^33^ (see M&M for details). The cells were maintained in the presence or absence of Dox from day 1 of differentiation and the eGFP expression was analyzed in the different hematopoietic populations at day 8 and day 15 (Fig. 7c). The CD45-CD34+ population (hemato-endothelial progenitors) were analyzed at day 8 (Fig. 7c, left panels), the more mature CD45+CD34- hematopoietic cells^33^,^49^ at day 15 (Fig. 7c, right panels) and the CD45+CD34+ cells (hematopoietic progenitors) were analyzed both days. Both Lent-On-Plus LVs showed very low leaking in the absence of Dox (compare Mock- with CEETnl2- and CEETnl2Is2- transduced cells in Fig. 7c) and good expression in its presence in all hematopoietic populations analyzed (CD45-CD34+, CD45+CD34+ and CD45+CD34-). The presence of the Is2 insulator in the CEETnl2Is2 had only a minor effect in the leaking of these populations compared to the CEETnl2-transduced cells (Fig. 7c, −Dox plots). Interestingly, the CEETnl2 outperformed the CEETnl2Is2 in terms of percentage of transduced cells, in part due to its better titer (Fig. 7c, +Dox plots, compare CEETnl2 versus CEETnl2Is2).

## Discussion

The development of technologies allowing conditional transgene expression in stem cells is fundamental not only to study gene function^2^ but also as potential tools for gene therapy^3^. However, conditional expression in human stem cells has been difficult to achieve due to the low efficiency of existing delivery methods, the strong silencing of the transgenes^50^ and the side effects of the regulators^13^,^14^,^15^,^16^,^17^,^51^,^52^,^53^. The Tet-ON has several interesting properties for its application in gene therapy^54^. However, current Dox-inducible systems have either, background expression in the absence of Dox, low sensitivity to Dox and/or complex establishment of inducible expression systems^9^,^55^.

We have previously described a Tet-On, all-in-one LV (CEST), based on the original TetR repressor that efficiently generates Dox-responsive cell lines^22^. However this system was unable to regulate transgene expression in pluripotent stem cells and required multiple vcn/c (22 and Fig. S2) to regulate transgene expression in 293 T and hMSCs. In this manuscript we report the second generation Tet-On all-in-one LVs systems (Lent-On-Plus) based on the original TetR repressor. The Lent-On-Plus LVs can generate dox-responsive human stem cells with only one vcn/c. Importantly, the regulation in stem cells is maintained over time in culture and upon differentiation toward different lineages.

Most Tet-On inducible systems are based on two components, one encoding the regulator (TetR, rtTA or its derivates) and a second one harboring the regulated promoter expressing the transgene (reviewed in^3^). However, these systems require antibiotic selection and/or cloning strategies to generate Tet-On cell lines. In addition they are very inefficient for *in vivo* applications due to the requirement to have both components in the same cell. Therefore, the design of a single vector expressing both the regulator and the transgene has become a priority for several applications. In this direction several authors have developed regulated all-in-one vectors that can modulate transgene expression^56^,^57^,^58^,^59^,^60^,^61^. However, these systems still required selection and/or cloning to obtain regulated pluripotent stem cells^5^,^9^ and, in addition, they used regulators consisting in protein chimeras harboring the VP16 transactivator (rtTA and derivates) which could be problematic for several applications because of squelching^13^,^14^,^15^ and/or activation of cellular genes due to binding of the chimeric TetR-VP16 protein to *pseudo-TetO* sites^16^,^17^. Contrary to these systems, our all-in-one LVs achieved good regulation of transgene expression in hESCs after one round of transduction without the requirement of any selection or cloning. In addition, the absence of transactivation domains in the regulator (TetRnl2) make the Lent-On-Plus systems potentially safer due to the lower influence on gene expression of transduced cells.

Several Tet-On all-in-one LVs based on the original TetR^62^ have been developed previously by several groups, including ours^22^. We have previously showed that TetR-based systems required high TetR levels for regulating the CMV-TetO promoter making it difficult to develop tightly regulated all-in-one LVs^22^. In our first generation all-in-one LVs (the CEST), we overcame this requirement by expressing the TetR repressor though the strong SFFV promoter^61^ and increasing the MOI to achieve 3–4 vector copies/cell. This strategy rendered Dox-responsive cell lines (293 T, hMSCs) expressing very high levels of transgenes with low concentrations of Dox but also having relative high leaking^22^. In a different approach, Ogueta *et al.*^38^ engineered regulated all-in-one LVs (named SIN-1PiN) by expressing the transgene through the Dox-regulated promoter (CMV-TetO) and the TetR repressor through an internal ribosomal entry site (IRES). Although this self-regulatory system enabled good transgene regulation in some cell types, the restricted cellular tropism of IRES elements limited its use for some applications. We (our data) and others^63^,^64^,^65^ have shown that the CMV-TetO promoter is highly methylated in stem cells precluding the application of these LVs (CEST and SIN-1PiN) in these cell types.

Therefore, in order to develop the next generation all-in-one Tet-ON regulatory system we needed to increase the repressor levels in the nuclei and to avoid silencing of the promoters expressing the transgene and the TetR. Here we have shown that we can increase the inducibility and reduce the leaking of the all-in-one LVs by incorporating a nuclear localization signal in the TetR (CESTnl2) and inserting the Is2 insulator^33^ (CESTnl2Is2). The insulators can shield integrative vectors from nearby regulatory domains favoring a more autonomous regulation. The Is2 element incorporates a synthetic SAR element and a HS4 insulator having two different effects in hESCs: 1- vector shielding and 2- enhancement of transgene expression^33^. Interestingly the CESTnl2Is2 LV expressed higher transgene levels in 293 T cells compared to CESTnl2, although this was not true for hMSC and hESCs. However, in spite of the improvements, the leaking of the CESTnl2Is2 system remained too high in 293 T and hMSCs. Moreover the CEST LVs did not regulate transgene expression in hESCs due to methylation of the SFFV promoter (Fig. 6). In agreement with our data, other groups have also showed SFFV silencing in pluripotent stem cells due to methylation^29^.

Our results also indicated that the expression of the TetRnl2 through the hEF1α promoter, instead of the SFFV promoter was a main determinant for the success of the Lent-On-Plus. Indeed, the presence of the hEF1α promoter (Lent-On-Plus LVs) reduced the leaking in all cell lines analyzed and, most importantly, allowed transgene regulation in hESCs and its progeny. Contrary to SFFV, the hEF1α promoter was previously shown to be highly resistant to methylation in pluripotent cells^34^,^46^,^47^. In agreement with these results, the hEF1α promoter in the Lent-ON-Plus LVs backbone was also resistant to methylation allowing a stable expression of TetR in hESCs. Another factor that could have a positive effect on the low leaking of the Lent-On-Plus LVs compared to the CEST is the lower activity of the CMV-TetO promoter in this backbone. A less active promoter could require less TetR molecules to block transcription and will have therefore lower leaking. The fact the CEETnl2Is2 LVs express lower levels of TetR than the CESTnl2Is2 in hMSCs (See Fig. S4) and yet, the CEETnlsIs2 presented lower leaking, point to this direction. However, there is another possible explanation to the lower leaking of the Lent-On-Plus compared to the CEST LVs: The strong activity of the SFFV promoter in MSCs could interfere with the binding of the TetR to the TetO sites avoiding transgene repression in the CEST LVs. We favor the hypothesis that both mechanisms are playing a role in the fold induction and leaking of Lent-On-Plus LVs and that the final outcome will depend on the activity of both promoters on the different cell lines.

As observed in the CESTnl2 backbone, the inclusion of the Is2 element in the CEETnl2 LVs further reduced leaking and improved fold induction of the CEETnl2 LVs in 293 T and hMSCs. However, although the Is2 insulator reduced the leaking of the Lent-On-Plus LVs in hESCs, we couldn´t detect any effect on fold induction. Interestingly, contrary to what was observed in the CESTnl2 backbone, the CEETnl2 and the CEETnl2Is2 LVs expressed similar eGFP levels in all cell lines. These data indicated that the effect of the Is2 was not only cell-dependent but was also backbone specific. Finally, although the presence of the Is2 insulator helps to improve the regulation of the all-in-one LVs, also reduced the titer of the vector 2–3 times. As consequence the transduction efficiency of the CEETnl2 is better than the CEETnl2Is2 and this difficult to reach high transduction efficiencies in hESCs (see Fig. 7).

Generation of regulatable human pluripotent stem cells has been an important goal for transgenesis and gene therapy. In a very elegant study, Qian *et al.* developed a strategy to generate inducible pluripotent stem cells based in specific integration of the regulatory cassette in the AAVS1 site^9^. Compared to this system, our all-in-one LVs have some advantages and disadvantages. The main limitation of our system compared to the strategy described by Qian *et al.* is the wider variability of expression levels between the regulated stem cells due to the random integration of the LVs. Importantly our system have also several advantages: 1- the possibility to generate regulated pluripotent stem cells without the requirement of selection or cloning, 2- The ability to generate inducible pluripotent stem cells with different expression levels by increasing the MOI or by multiple rounds of transduction and 3- Our vectors could also be used for *in vivo* applications, including gene therapy strategies

Absence of expression in the absence of dox could be due to tight regulation or to other reasons, such as silencing or transcriptional variegation. Our data indicates that, in regulated 293 T and hMSCs (−Dox), the absence of expression is mainly due to TetR repression since we found a good correlation between the percentage of cells expressing eGFP (in the presence of Dox) and the vcn/c. However, in hESCs, most integrated LVs do not express eGFP even in the presence of Dox. Indeed, only 20% of the hESCs express eGFP in LVs–transduced hESCs harboring 1–2 vcn/c. This data is in accordance with previous publications showing that only a fraction of integrated retroviral vectors express the transgene in ES^66^. Other authors have found that proviruses at different integration sites display variegation^67^, expressing the transgene in some cells but not in others. We favor the later hypothesis to explain the heterogeneity of expression observed in bulk hESCs populations. Therefore, in hESCs not all integrated LVs will be able to respond to Dox at a certain time point and this is probably the cause of the low expression levels and the low sorting efficacy for the CEETnl2Is2-transduced population in Fig. 5. Interestingly, differentiation of transduced-hESCs toward the mesenchymal or the hematopoietic lineages increased the percentages of Dox-responsive cells compared to undifferentiated cells. We do not have an explanation for these increments but this data shows the stability of the Lent-On-Plus system to maintain Dox-responsiveness in transduced-hESCs upon culture and differentiation. Although the CMV promoter is probably not the best choice for transgene expression in stem cells, we hypothesized that it could be a good choice to achieve widespread transgene regulation in hESC-derived cells. Our results showed that, in the Lent-On-Plus backbone, the CMV-tetO promoter can achieve stable and modest expression in hESCs and hESCs-derived hematopoietic cells and good expression level in hESCs-derived MSCs. We are currently modifying Lent-On-Plus configuration to include a panel of constitutive and tissue-specific TetO promoters to extend the applications of this system.

*In vitro* differentiation of hESCs toward the hematopoietic lineage provides a unique tool to study human hematopoietic development, as a platform for drug screening and as a potential source for cell-gene therapy strategies^68^,^69^,^70^. To our knowledge, this is the first report of a Tet-ON system that achieves transgene regulation in hematopoietic cells derived from pluripotent stem cells. It is noteworthy to mention the low leaking and good expression levels of the CEETnl2 in all hematopoietic cells analyzed. Indeed, this LVs vector was able to maintain high transgene expression levels (over 90% in some cell types) and good inducibility in the absence of the Is2 insulator.

In summary, we have developed three new Tet-On all-In-One LVs systems with a wide range of applications. Two of them, the CEETnl2 and the CEETnl2Is2 (the Lent-On-Plus system) represent, to our knowledge, the first all-in-one LVs able to generate Dox-responsive human pluripotent stem cells after one single round of transduction, without requirement of selection and/or cloning. This is also the first report of a Tet-ON system that achieves transgene regulation in hematopoietic progenitors and mature cells derived from unselected pluripotent stem cells. Finally, the different all-in-One LVs developed in this work can fulfill different requirements for different applications. The CESTnl2Is2 can be use for differentiated and immortalized cell lines and when the main objective is to achieve high transgene levels upon Dox addition (for example for production of toxic proteins in cellular factories). The CEETnl2 LV is the best system for the generation of dox-responsive pluripotent stem cells and when efficiency is preferred versus very-low leaking. Finally the CEETnl2Is2 LV is the best choice when a very low leaking is required.

## Material and Methods

### Plasmids construction

Two different nuclear sequences named nl1 and nl2 were incorporated at the 3′end of TetR preceding the stop codon in the StetR LV^22^ to generate STetRnl1 and STetRnl2. nl1 consist in a three tandem repeat sequence corresponding to the nuclear localization signal (DPKKKRKV) from SV40, whereas nl2 (RKCLQAGMNLEARKTKK) is the nuclear localization signal from the glucocorticoid receptor. A PstI-PstI 1231 bp fragment from the STetRnl1 and STetRnl2 LVs was inserted within the unique PstI site of the CEWP vector^71^ to get the CESTnl1 and CESTnl2 respectively. CESTnl2Is2 were constructed by inserting a KpnI-NheI 1509 bp fragment from the SE-Is2Rev^33^ into the KpnI-NheI site of CESTnl2. CEETnl2 and CEETnl2Is2 were constructed in three steps: 1- a DNA fragment encompassing the TetRnl2 sequence was amplified from the StetRnl2 and inserted into the pLVTHM LV (Addgene plasmid 12247; http://www.addgene.org/12247) using standard molecular techniques to obtain the pLVTHM-TetRnl2. 2- The CEETnl2 vector was generated by insertion of a SalI-KpnI fragment (encompassing the hEF1α-TetRnl2) into the PstI site of CEWP. 3- Finally, the CEETnl2Is2 was constructed by inserting the kpnI-NheI 1509 bp fragment from the SE-Is2Rev^33^ into the KpnI-NheI site of CEETnl2.

### Lentiviral vector production, cells transduction and calculation of vector copy number per cell

The human immunodeficiency virus (HIV) packaging (pCMV_R8.91) and VSV-G (pMD2.G) plasmids (http://www.addgene.org/Didier_Trono) are described elsewhere^72^,^73^. Vector production was performed as previously described (Benabdellah *et al.*^22^). Briefly, 293 T cells were plated on amine-coated petri-dishes (Sarsted,Newton, NC). The vector (CESTnl1, CESTnl2, CESTnl2Is2, CEETnl2 or CEETnl2Is2), the packaging (pCMVΔR8.9) and the envelope (pMD2.G) plasmids were transfected into the 293 T cells using LipoD293 (SignaGen, Gainthersburg, MD, USA). Viral supernantants were collected and frozen or concentrated by ultrafiltration at 2000 g and 4 °C, using 100 Kd centrifugal filter devices (Amicon Ultra-15, Millipore, Billerica, MA)^24^. Freshly collected virus particles, were used to transduce hMSCs and hESC as previously described^22^,^26^. Briefly, hMSCs were dissociated and mixed with the viral particles at the desired MOI, left at room temperature for 10 minutes, seeded and maintained at the incubator (5%O2; 5%CO2; 37 °C and humidity) for 5 hours. hMSCs were then washed and seed in a bigger flask for expansion. hESCs were dissociated with collagenase type IV and plated on fresh matrigel in the presence of the fresh viral particles. The media was changed after 5 hours. When the colonies reached confluence they were split and expanded in fresh matrigel tissue flasks.

The vector copy number per cell of transduced cells was calculated using 0.6 μg of genomic DNA ( = 100,000 hESCs) and plasmid DNA (from 10^2^ to 10^7^ copies) for the standard curve. The Q-PCR was performed as previously described^36^ using the eGFP primers shown in Supplementary Table 1.

### Cell Lines and Culture Media

293 T cells (CRL11268; American Type Culture Collection; Rockville, MD) were grown in Dulbecco’s Modified Eagle Medium (DMEM, Invitrogen, Edinburgh, Scotland) with GlutaMAX™ and supplemented with 10% Fetal Bovine Serum (FBS) (PAA Laboratories GmbH, Austria). hMSCs were obtained from Biobanco del Sistema Sanitario Público de Andalucía (Granada, Spain) and cultured as previously described^74^. Briefly hMSCs were cultured in advanced Dulbecco’s modified Eagle’s medium (DMEM) supplemented with 10% fetal calf serum (FCS) (Invitrogen, Carlsbad, CA, www.lifetechnologies.com), Glutamax (GIBCO, Grand Island, NY, www.lifetechnologies.com), and 100 U/ml penicillin/streptomycin (GIBCO). All experiments using human samples were performed according to the Institutional Guidelines and approved by the ethics committee of the H.U. Virgen de Macarena. AND-1 (Spanish Stem Cell Bank. www.isciii.es)^75^ and H9 (Wicell Research Institute Inc. Madison, WI) hESC lines were maintained undifferentiated in a feeder-free culture as previously described^26^. Briefly hESCs were culture in matrigel-coated T25 flasks and fed daily with hMSC-conditioned medium^70^ supplemented with 8 ng/ml bFGF (Miltenyi Biotech, Bergish Gladbach, Germany). Approval from the Spanish National Embryo Ethical Committee was obtained to work with hESCs.

### Adipocyte and Osteocyte differentiation

Untransduced hMSCs as well as CEETnl2- and CEETnl2Is2 transduced hMSC were differentiated to adipocytes and osteocytes as previously described^76^. Briefly, hMSCs were plated in 6-well plates at a density of 20,000 cells/cm^2^ for adipogenesis, 10,000 cells/cm^2^ for osteogenesis and incubated in the adipogenic and osteogenic MSCs differentiation BulletKits respectively (lonza, Basel, Switzerland).

### Human ESCs hematopoietic differentiation in OP9 co-culture system

Hematopoietic differentiation was induced as described by Ji *et al.*^49^. Briefly, the hESCs lines were transferred onto OP9 feeders for 15 days. To evaluate hematopoietic differentiation and eGFP expression at the different days, cells were dissociated with collagenase IV and Tryple (Gibco), resuspended in FACS buffer, filtered through a 70 μm cell strainer (BD Biosciences, Bedford, MA) and stained with anti-mouse CD29-FITC (AbD Serotec, Raleigh, GBK), anti-human CD34-PE-Cy7 and anti-human CD45-APC (all from eBiosciences, San Diego, CA) and analyzed in a FACS Canto II flow cytometer.

### Differentiation of hESCs toward the mesenchymal lineage

Derivation of MSC-like cell was carried out as previously described^77^, Briefly, AND-I hESC were treated with 10 μM ROCK for 1 hours, and disaggregated into a single cells with trypsin. The cell were collected in maintenance medium (Embryonic fibroblast conditioned medium supplemented with 4 ng/ml FGF) (Invitrogen), and seeded at a density of 15000/cm2 in Type I collagen coated well tissue culture. 24 hours later, the maintenance medium was supplemented with an equal volume of basal α-MEM (invitrogen) with 10% FBS (Hyclone), 100 U/ml penicillin, 100 μg/ml streptomycin (Invitrogen) and 50 μM magnesium L-ascorbic acid phosphate (Sigma-Aldrich). 10 days later, the medium was replaced with α-MEM (Invitrogen), supplemented with 100 U/ml penicillin and 100 μg/ml streptomycin, 2 mM L-glutamine. The third passage was used for flow cytometry analysis.

### FACS analysis and FACS-sorting

#### eGFP expression of total populations

The different cell types cultured in the presence of absence of Dox (0.1 μg/ml) were resuspended in PBS+FBS+EDTA buffer (FACS buffer) and filtered through a 70 um cell strainer (BD Biosciences, Bedford, MA). Live cells identifier by 7-AAD viability dye exclusion were analyzed for eGFP expression using a FACS Canto II flow cytometer equipped with the FACS Diva analysis software (BD Bioscience).

#### eGFP expression in hESCs-derived subpopulations

hMSCs derived from untransduced and transduced hESCs were resuspended in FACS buffer and stained with anti-CD105-APC antibodies (eBioscience, San Diego, CA) and analyzed by FACS. eGFP expression was measured in the CD105+ population. Similarly, hematopoietic cells derived from untransduced and transduced hESCs were resuspended in FACS buffer and stained with anti-CD34-PE-Cy7 (BD Biosciences) and anti-CD45-APC (Miltenyi Biotech, Bergish Gladbach, Germany) antibodies. Live cells identified by 7-AAD viability dye exclusion were analyzed for surface-marker and eGFP expression using a FACS Canto II. The populations analyzed were hemogenic progenitors (CD45^**−**^CD34^+^), hematopoietic progenitors (CD45^+^CD34^+^), and mature blood cells (CD45^+^CD34^−^).

#### Analysis of pluripotency markers in untransduced and transduced hESCs

Untransduced and transduced hESCs were stained with the antibodies oct3/4 (BD Pharmingen), seea-3, tra-1–60, tra-1–81 (eBiosciences) and live cells identified by 7-AAD viability dye exclusion (eBiosciences)(data not shown). Samples were acquired and analyzed in a FACS Canto II flow cytometer equipped with the FACS Diva analysis software (Becton Dickinson, Franklin Lakes, NJ).

Separation of eGFP+ hMSCs and hESCs. Dox-induced eGFP+ populations of CEETnl2 and CEETnl2Is2-transduced hMSCs or hESCs were washed and separated using fluorescence-activated cell sorting (FACS) Aria II Flow Cytometer. A MOI = 0.4 was used to keep the percentage of transduced hMSCs below 15%. For hESCs a MOI = 30 was used to achieve over 30% eGFP+ cells and good expression levels for sorting.

### Western Blot

Cells were lysed in RIPA buffer (Sigma) containing protease inhibitors (Sigma Aldrich) at 1 × 10^6^ cells/100 μl. The lysates (20 μg/sample) were resolved by sodium dodecyl sulphate-polyacrylamide gel electrophoresis (10% polyacrylamide under reducing conditions) and electrotransferred to nitrocellulose membranes (Bio-Rad Hercules CA.). Milk-blocked Membranes were probed for 1 hr at room temperature with mouse anti-Tet-repressor (Mobitec; no TET02) at 1:500 dilution at 4 °C, and rabbit anti-tubulin (Santa Cruz, sc-5546). Combination of IRDye 680LT Goat anti-Rabbit IgG (Licors: no 26-68021) and IRDye 800CW Goat anti-Mouse IgG (Licors: no 926-32210) were use at 1:10.000 dilutions to analyzed TetR and tubulin using an Odyssey Image scanner system (LI-COR Biosciences).

### Determination of fold induction and leaking index

Transduced cells incubated in the presence or absence of Dox (0.1 μg/ml) were analyzed by flow cytometry to determine the percentage and Mean fluorescence intensity (MFI) of eGFP positive cells as well as the MFI of WLC (WLC = P1 defined as the 7-AAD negative population). The fold induction was calculated using the following formula MFI P1 (+Dox)/MFI P1 (−Dox). Similarly, the *Leakiness* of the system was determined by using the following formulas [% eGFP^+^ (−Dox) x 100/%eGFP^+^(+Dox)] or [[% eGFP^+^ (−Dox) x 100/%eGFP^+^(+Dox)]] x MFI eGFP+(−Dox) (supplementary information). Background (%eGFP^+^ cells in MOCK transduced cells) were subtracted to the % eGFP^+^ obtained in the different transductions and conditions. Therefore when the %eGFP+ (−Dox) is identical in Mock and Transduced cells the leaking is zero and when the %eGFP+ (−Dox) is identical to the %eGFP+(+Dox) the leaking is 100.

### DNA methylation assay

The promoter methylation was assessed by bisulfite treatment of Genomic DNA and sequencing of the resulting converted gDNA. In the presence of sodium bisulfite, only the unmethylated cytosines are chemically converted to uracil. The genomic DNA was isolated from hESCs at the desired time points using the DNaseasy kit (Qiagen, Crawly, UK). Sodium bisulfite treatment of genomic DNA was performed using the EpiTect bisulfit kit (Qiagene), according to the manufacurer’s instruction. The converted gDNA was used for a nested PCR amplification of SFFV and EF1a promoters. The primers used for each amplification is shown in Suplementary Information (Table S1). The PCR product band was purified and cloned into PCR 2.1 (Invitrogene) according to the manufacturer′s instruction. 15–30 clones were randomly selected and sequenced with M13 primers. Identical sequences were eliminated from the study to avoid duplicated clones.

### Statistical Analysis

All data are represented as mean +/− (SEM). The statistical analysis was performed using the GraphPad Prism software (GraphPad Software, Inc., La Jolla, CA, www.graphpad.com) applying the unpaired two-tailed t-test. Statistical significance was defined as a *P* value < 0.05.

## Additional Information

**How to cite this article**: Benabdellah, K. *et al.* Lent-On-Plus Lentiviral vectors for conditional expression in human stem cells. *Sci. Rep.* **6**, 37289; doi: 10.1038/srep37289 (2016).

**Publisher’s note**: Springer Nature remains neutral with regard to jurisdictional claims in published maps and institutional affiliations.

## Supplementary Material

Supplementary Information
