# Supplementary material for: Lent-On-Plus Lentiviral vectors for conditional expression in human stem cells
Source: Sci Rep. 2016 Nov 17;6:37289. doi: 10.1038/srep37289 (PMC5112523; doi:10.1038/srep37289)
Supplement: Supplementary Information [file srep37289-s1.pdf]

Supplementary Information

Lent-On-PluS Lentiviral vectors for conditional expression in human stem cells.

Karim Benabdellah[1,5,*,$], Pilar Muñoz[1,2,$], Marién Cobo[1], Alejandra Gutierrez-Guerrero[1], Sabina Sánchez-Hernández[1], Angélica Garcia-Perez[1,3], Per Anderson[1], Ana Belén Carrillo-Gálvez[1], Miguel G. Toscano[1,4,] and Francisco Martin[1,5,*]

[1]Genomic Medicine Department. GENYO, Centre for Genomics and Oncological Research, Pfizer-University of Granada-Andalusian Regional Goverment, Parque Tecnológico Ciencias de la Salud, Av. de la Ilustración 114, 18016 Granada, Spain.

[2] Current address: University College London-Institute of Child Health, 30 Guilford Street. WC1N 1EH. London, United Kingdom

[3]Current address: Max-Delbrück-Center for Molecular Medicine, Berlin, Germany

[4] Current address: Amarna Therapeutics S.L., Marie Curie Building, C/ Leonardo da Vinci 18ª. Isla de la Cartuja, 41092 Seville, Spain

[5] LentiStem Biotech. GENYO, Centre for Genomics and Oncological Research, Pfizer-University of Granada-Andalusian Regional Goverment, Parque Tecnológico Ciencias de la Salud, Av. de la Ilustración 114, 18016 Granada, Spain.

[$] These author contributed equally to this work

[*]Correspondence to Francisco Martin: francisco.martin@genyo.es or Karim Benabdellah: karim.benabdel@genyo.es

Supplementary Figures

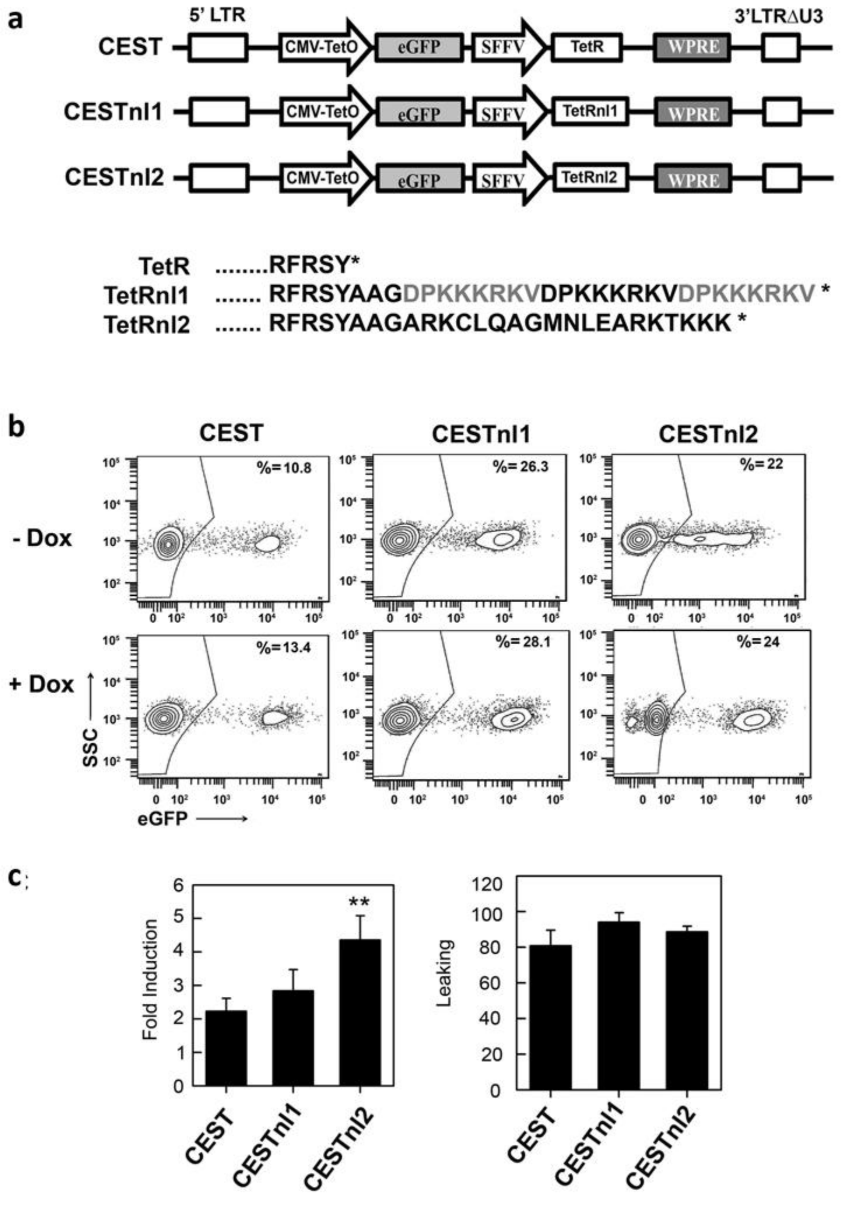

Figure S1 Introduction of nuclear localization signals increase the fold induction of the CEST LV. (a) Schematic representation of CEST, CESTnl1 and CESTnl2 lentiviral vectors (LVs) (top) and the NH3 terminus of the TetR, TetRnl1 and TetRnl2 (bottom). The TetRnl1 contain a linker peptide (AAG) and three tandem repeats of the nuclear localization signal of the SV40 T antigen (DPKKKRKV). In the TetRnl2 we included the same linker peptide and the nuclear localization signal of the glucocorticoid receptor (ARKCLQAGMNLEARKTKKK). (**b**) Representative plots showing eGFP expression of 293T transduced with different construct (CEST, CESTnl1, CESTnl2) in the absence (top) or presence (bottom) of Doxycycline (Dox). (**c**) Graphs showing fold induction (left) and leaking (right) of the different LVs in 293 T. Values

represent mean +/- standard error of the mean of at least three separate experiments (**p<0.01)

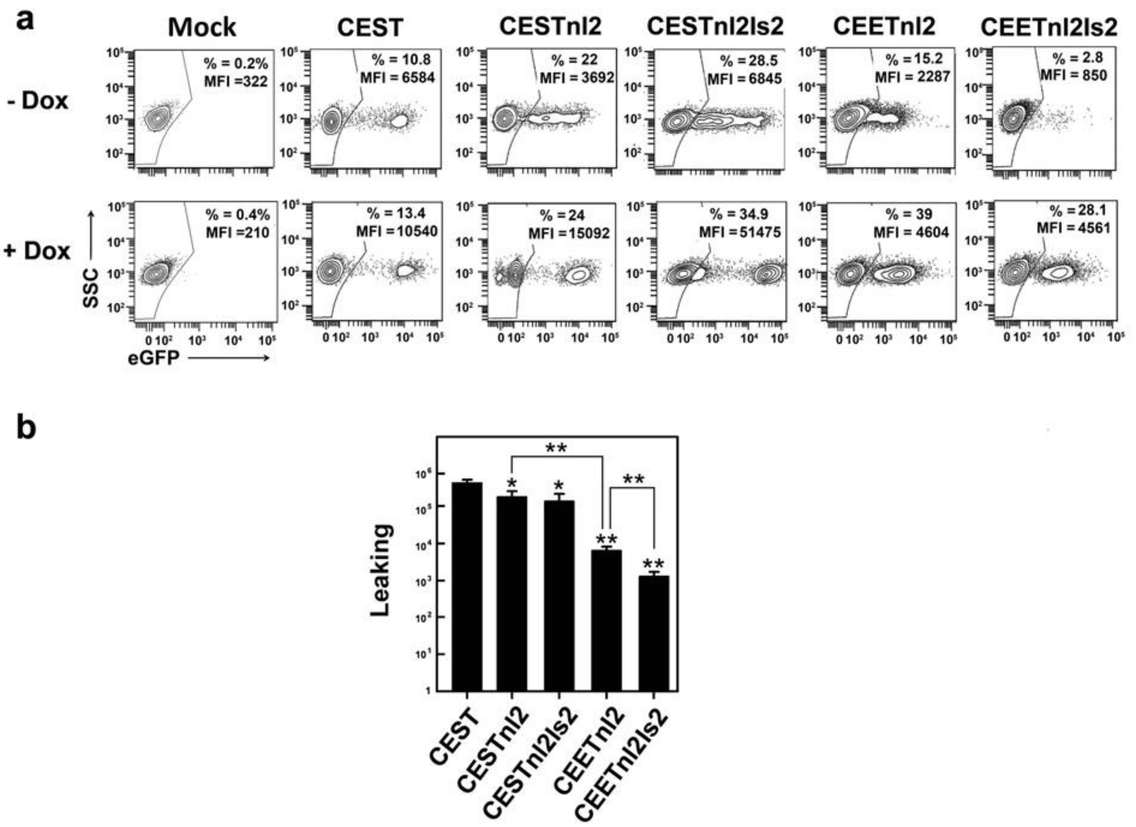

Figure S2. ***Leaking of the different LVs in 293T using an alternative analysis*.** (**a**) Representative plots showing eGFP expression profiles of untransduced 293T (Mock) and 293T cells transduced with the different LVs (as indicated at the top of each plot) in the absence (top) or presence (bottom) of Dox (0.1µg/ml). A MOI = 0.3 was used to keep the percentage of eGFP+ cells below 30% (in order to keep transduced cells with only one LV integration). The gates of the eGFP+ populations were set to 0.2-0.4% of eGFP+ cells in the untransduced population (Mock; left plots) and subtracted to the % obtained under the different vectors and conditions for the analysis. The percentage (%) of the eGFP+ population (used to measure leaking) and the Mean Fluorescence Intensity (MFI) of the eGFP+ population are shown in each plot (**b**)**.** Graph showing leaking of the different LVs in 293T taking into account the MFI of the eGFP+ population in the absence of Dox: (Leaking = [[%eGFP+(-Dox) *100/%eGFP+(+Dox)] * MFI eGFP+ (-Dox)]. To measure leaking, the background (% of eGFP+ of Mock cells) were subtracted to the % of the eGFP+ under the different conditions. Values represent mean +/- standard error of the mean of at least four separate experiments Asterisks indicate significance related to CEST (on top of the bars) or significance between the CEETnl2 and CEETnl2Is2 (as indicated in the Figure)(* p<0.05; ** p<0.01).

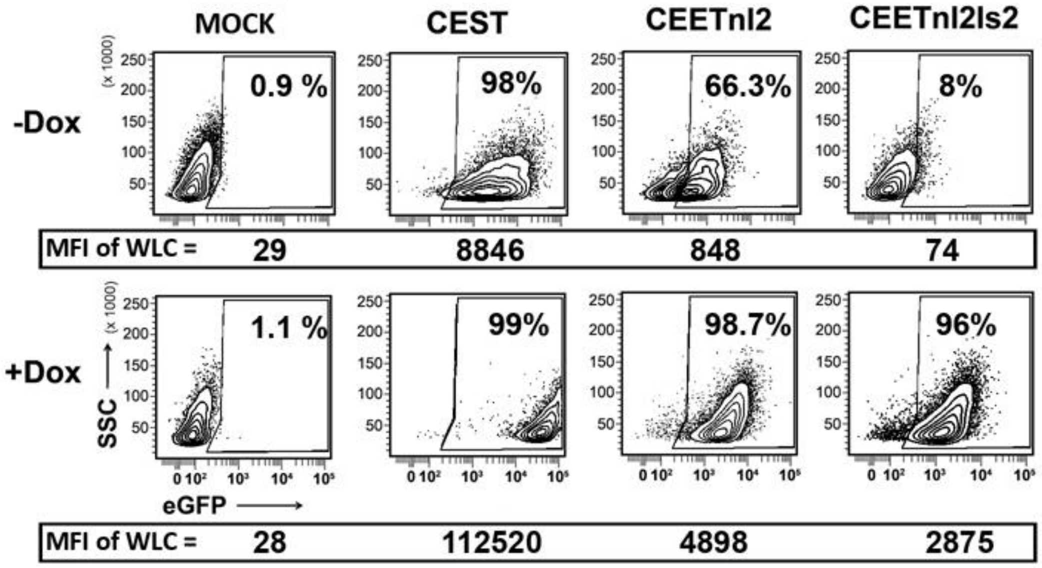

	CEST	CEETnl2	CEETnl2Is2
Fold Induction	12.7	5.7	38.8
Leaking	98	66.2	7.4

Figure S3. ***The CEETnl2Is2 LV achieved high fold induction and low leaking of on 293T transduced at high MOI.*** Top: plots showing eGFP expression of untransduced (Mock) and CEST- CEETnl2- and CEETnl2Is2- transduced 293T cells in the absence (top) or presence (bottom) of Doxycycline (Dox). The MFI of whole living cells (WLC) are shown at the bottom of each plot. Fold induction and leaking of the different transduced populations are indicated in the table (bottom).

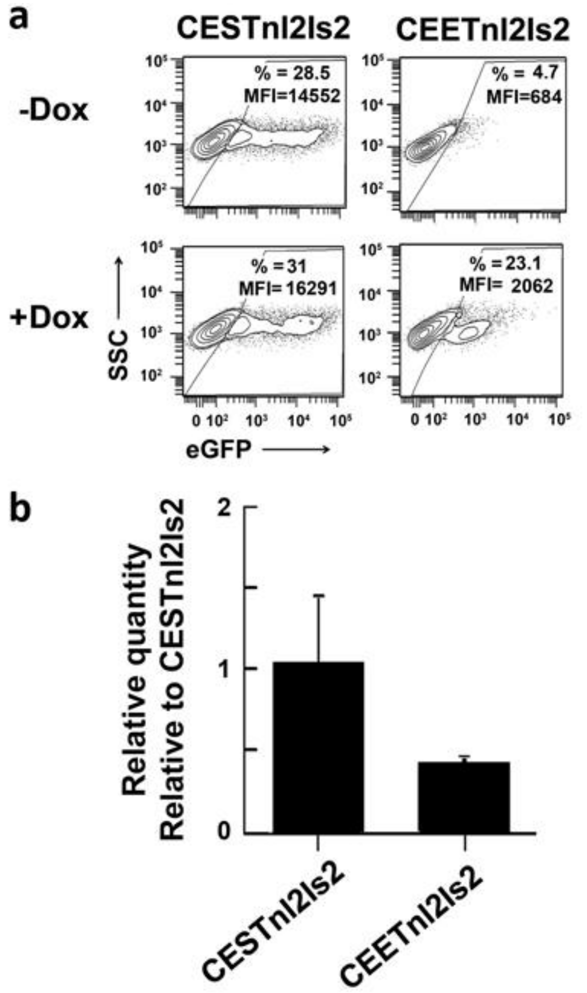

Figure S4. Analysis of the TetR expression levels of the different LVs backbones. (a) Regulation of CESTLN2IS2 and CEETLN2Ins2 in human Mesenchymal Stem Cells (hMSCs). Representative plots showing eGFP expression profiles of hMSCs transduced with CESTnl2Is2 and CEETnl2Is2 LVs in the absence (top) or presence (bottom) of Dox. The percentage (%) and mean fluorescence intensity (MFI) of the eGFP+ population are shown in each plot. (**b**) Relative expression of the TetR mRNA CSETnl2- and CEETnl2-transduced hMSCs. TetR gene expression was assessed by RT-qPCR (see M&M for details). Samples were normalized using the housekeeping gene Gadph. Relative expression levels were calculated by the 2-ΔΔCT method normalized to the CESTnl2 data. Data are means +/- standard error

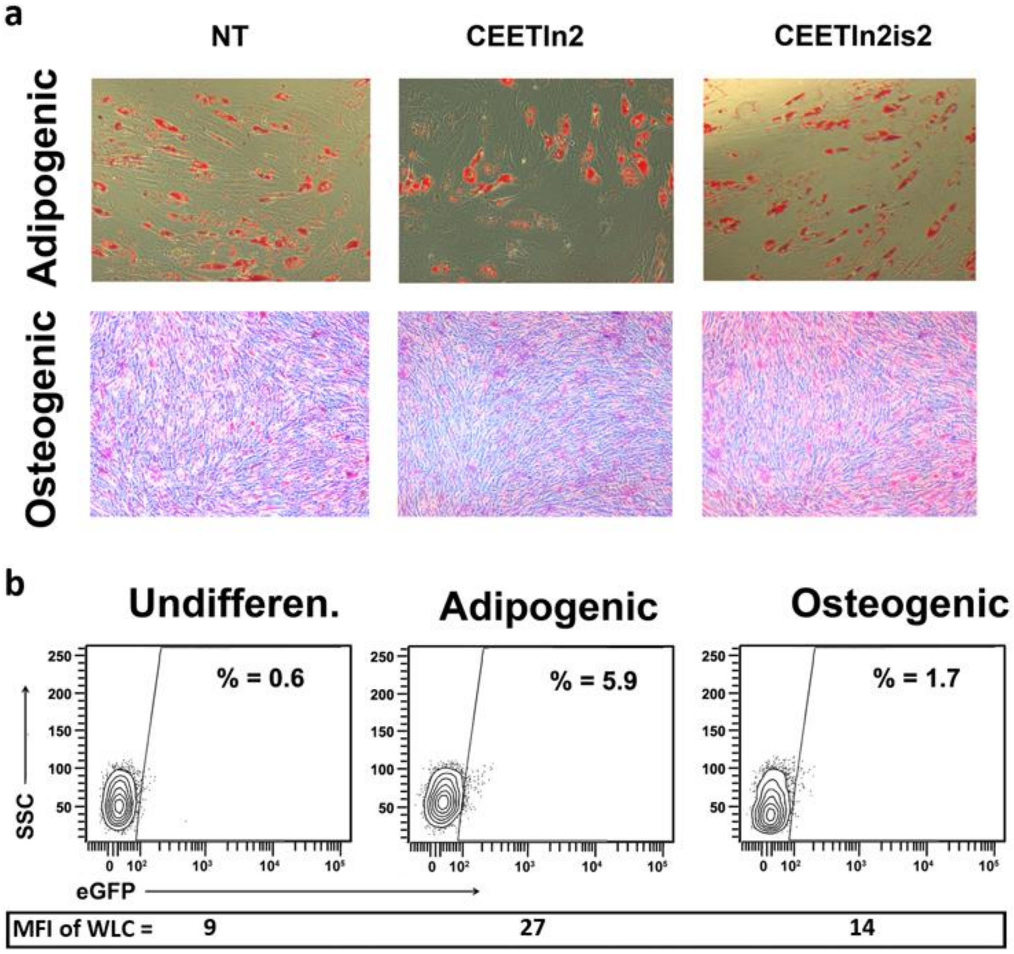

Figure S5. Analysis of multipotency of transduced hMSCs and analysis of autofluorescence of the untransduced populations. (a). Untransduced (Mock), CEETnl2- and CEETnl2Is2-transduced hMSCs were differentiated toward the adipoogenic (top panels) or osteogenic (bottom panels) and stained with oil red and Alizarin Red S respectively (See supplementary Methods for details). (**b)** Representative plots of the background fluorescence of the undifferentiated MSCs (left), adipogenic (middle) and osteogenic (reight) cells. The MFI of whole living cells (WLC) are shown at the bottom of each plot.

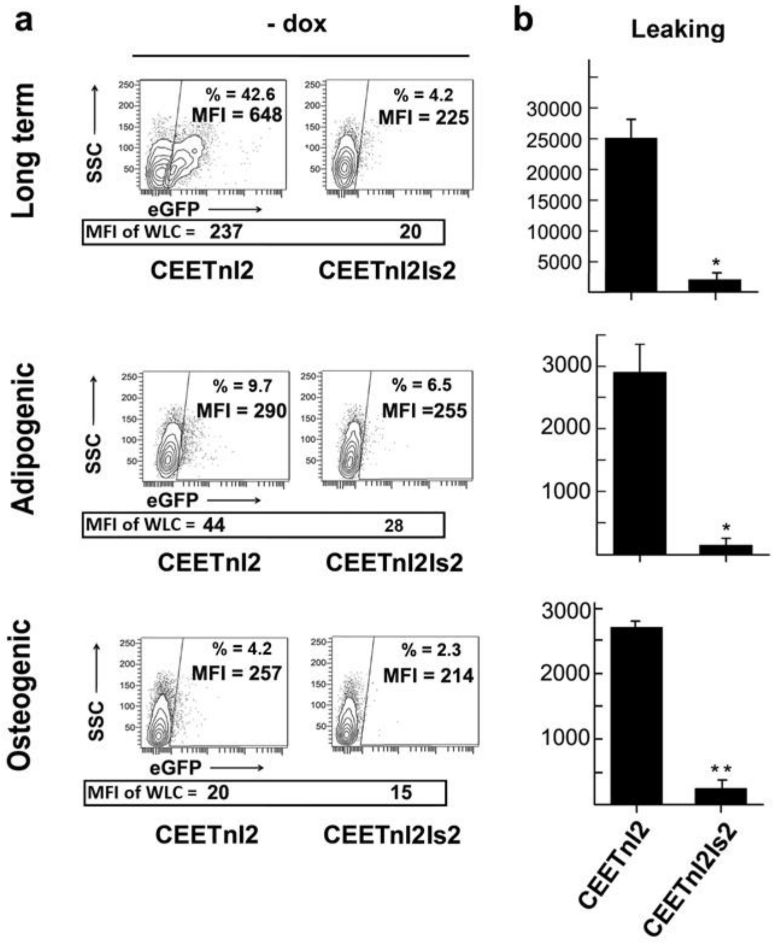

Figure S6. Leaking of the CEETnl2 and CEETnl2Is2 in MSCs using an alternative analysis. (a) Representative plots showing eGFP expression profiles of hMSCs transduced with CEETnl2 and CEETnl2Is2 LVs in the absence of Dox after 40 days in culture (long term) and after differentiation toward the adipogenic (middle plot) and osteogenic (bottom plots) lineages. The gates of the eGFP+ were set to have 0.6%, 5,9% and 1.7% of eGFP+ cells in the untransduced population of the long term, adipogenic and osteogenic cells respectively (Fig. S5). The percentage (%) and the MFI of the eGFP+ population are shown in each plot (**b**) Graphs showing **l**eaking taking into account the MFI of the eGFP+ population in the absence of Dox: (Leaking = [[%eGFP+(-Dox) *100/%eGFP+(+Dox)] * MFI eGFP+ (-Dox)]. To measure leaking, the background (% of eGFP+ of Mock cells) were subtracted to the % of the eGFP+ under the different conditions. Values represent mean +/- standard error of the mean of at least three separate experiments (* $p<0.05$; ** $p<0.01$)

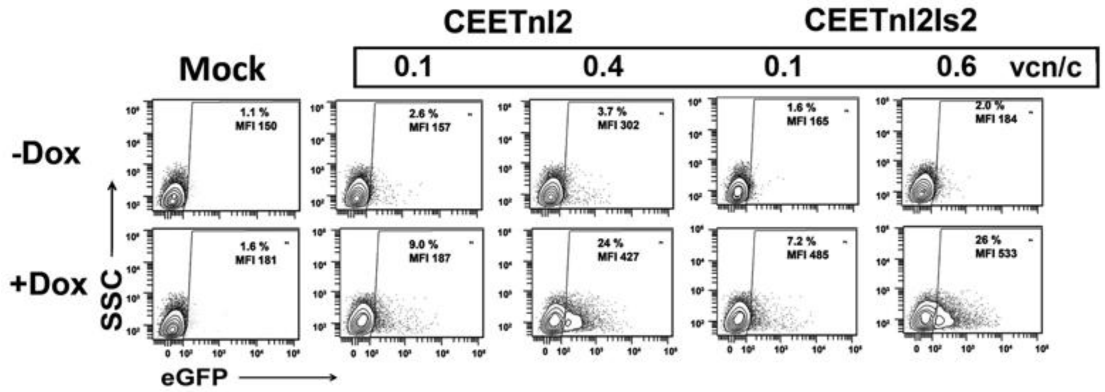

Figure S7. LentOnPlus LVs regulate transgene expression in hESCs at low vcn/c. Representative plots showing eGFP expression profiles of hESCs (AND-1) transduced with CEETnl2 and CEETnl2Is2 LVs in the absence (top) or presence (bottom) of Dox. hESCs were transduced at two different MOIs for each vector (MOI=0.3 and MOI = 1) in order to achieve a low transduction level. The percentage (%) and mean fluorescence intensity (MFI) of the eGFP+ population were analyzed by flow cytometry.

Table S1

**SFFVf1**	**5´-TAGAAAAAGGGGGAATGAAA– 3’**
**eGFPr1**	**5´- AAACAACTCCTCACCCTTACTCAC– 3’**
**SFFVf2**	**5´- GGGGGAATGAAAGATTTTATTTG– 3’**
**eGFPr2**	**5´- CCCTTACTCACCATAATTTCAAC C– 3’**
**EF1αf1**	**5´- GATGGATAAAGTTTTAAAT– 3’**
**EF1αr1**	**5´-CTATTCTTTCCCCTACACTATAC– 3’**
**EF1αf2**	**5´-AAGTTTTAAATAGAGAGGAA– 3’**
**EF1αr2**	**5´-CTACACTATACCCCCCAATCC– 3’**
**TetRf**	**5´-GGGATCCTAGTGATTATGTCT– 3’**
**TetRr**	**5´-TTACGGGTTGTTAAACCTTC– 3’**
**GADPHf**	**5´-GAAGGTGAAGGTCGGAGTC– 3’**
**GADPHr**	**5´-GAAGATGGTGATGGGATTTC– 3’**
**eGFPfw**	**5´- GTTCATCTGCACCACCGGCAAG-3´**
**eGFPrev**	**5´- TTCGGGCATGGCGGACTTGA-3´**

Supplementary Table 1. Primers used for the methylation profile analysis and determination of vector copy number per cell (vcn/c).

Supplementary Methods

Analysis of the Adipocyte and Osteocyte differentiation:

The different cells line were incubated in osteogenic and adipogenic MSCs differentiation BulletKit media respectively (lonza, Basel, Switzerland), and stained with Oil red (Amresco, solon, OH) to stain lipid vesicles present in adipocytes and with Alizarin Red S (Sigma) to stain calcium deposits in osteocytes

mRNA analysis by RT-qPCR.

Total RNA from hMSCs transduced with CESTln2Is2 or with CEETLN2Is2 was isolated using the Trizol reagent (Invitrogen) and reverse-transcribed using the Superscript first-strand system (Invitrogen). qPCRs were performed using the QuantiTect SYBRGreen PCR kit (Qiagen) on a Stratagene MX3005P system (Agilent Technologies, Santa Clara, CA). Q-PCR reactions consisted of 40 cycles at 94 ºC (15 sec), then 60 ºC (30 sec) and 72 ºC (30 Sec). TetR and GADPH specific primers are shown in Table S1. The relative expression was calculated using ΔΔCT method (Livak and Schmittgen 2001).
